# Insights Into Chikungunya Virus Seropositivity and Clinical Manifestations During the 2024 Upsurge: A Study From Central India

**DOI:** 10.7759/cureus.115672

**Published:** 2026-09-02

**Authors:** Vishal Shete, Meena Mishra, Omna Nelumbium, Rashmi Mudliar, Bharatsing D Rathod, Nishaant Ramasamy, Preeti A Shahu, Shailendra Parihar

**Affiliations:** 1 Microbiology, All India Institute of Medical Sciences, Nagpur, Nagpur, IND; 2 General Medicine, All India Institute of Medical Sciences, Nagpur, Nagpur, IND; 3 Community Medicine, All India Institute of Medical Sciences, Nagpur, Nagpur, IND

**Keywords:** central india, chikungunya virus, clinical manifestations, complications, seropositivity

## Abstract

Background

Chikungunya remains a significant threat to public health in India, characterised by repeated resurgences. This study was conducted to describe the demographic and clinical profile and associated complications among chikungunya patients during the 2024 upsurge.

Methods

This retrospective observational study included patients with acute febrile illness who tested positive for chikungunya immunoglobulin M (IgM) using enzyme-linked immunosorbent assay (ELISA) between January and December 2024. Demographic data, symptoms, comorbidities, co-infections, and complications were retrieved from institutional records. Statistical analysis was done using the chi-square test.

Results

A total of 943 patients were tested for chikungunya virus (CHIKV), of which 117 (12.4%) were positive. The majority were adults (19-49 years, 61.5%) and urban residents (60.7%). Arthralgia was the most common symptom, predominantly affecting large joints (65.8%) compared to small joints (50.4%). Rash was noted in 26.5% of cases. Complications occurred in 43.6% of patients. Among these complications, the most common was persistent arthralgia (13.7%).

Conclusion

The seropositivity study of the CHIKV upsurge has provided valuable insights into the symptomatology and complications associated with chikungunya infection. Chronic arthralgia for more than three months is the most common complication seen. Although studies have described greater involvement of small joints than large joints, in our case, we have reported a higher number of large-joint involvements in chikungunya.

## Introduction

Chikungunya virus (CHIKV) is an alphavirus transmitted primarily by *Aedes aegypti* and *Aedes albopictus* mosquitoes. It was first detected in 1952 during a significant episode of febrile arthritis in southern Tanzania and has since emerged as a major public health concern worldwide [1]. Four genotypes have been identified: East-Central-South African (ECSA), West African, Asian, and the Indian Ocean Lineage [2]. The disease usually presents with a sudden onset of fever accompanied by joint pain, headache, muscle aches, nausea, rash, and fatigue. Although chikungunya infection is often self-limiting, approximately 30-40% of affected individuals experience chronic polyarthralgia. In recent times, CHIKV have been associated with diverse complications and increased rates of morbidity and mortality [3].

Clinical presentations vary widely but typically include the classical triad of fever, arthralgia/arthritis, and rash [4]. Ocular symptoms (such as conjunctivitis, retro-orbital pain, etc.) may also occur and usually resolve within six to eight weeks. Notably, acute CHIKV infection closely resembles that of other arboviral illnesses, such as dengue and Zika, often leading to misdiagnosis in co-endemic regions [5]. However, unlike dengue and Zika virus infections, which are symptomatic in roughly 20% of cases, approximately 85% of individuals infected with CHIKV develop symptomatic illness [5].

India has experienced several CHIKV outbreaks, with a major resurgence occurring in 2006 after a hiatus of over three decades. This outbreak began in the southern states and spread rapidly to other regions, including central India. Maharashtra was significantly affected, reporting over 2,00,000 suspected cases [6].

In 2024, Central India saw a surge in cases, with Maharashtra among the worst-affected states. According to the National Centre for Vector-Borne Disease Control (NCVBDC) [6], confirmed cases in India in 2024 totalled 17,930, of which Maharashtra alone accounted for 5,854, which is more than a threefold increase compared with 2023 (1,702 confirmed cases in Maharashtra) [6]. This study aims to assess key symptoms and demographic characteristics during the 2024 CHIKV infection upsurge, and secondary objectives include assessing the frequency of complications.

## Materials and methods

Study setting

This is a retrospective observational study conducted at a tertiary care centre in Nagpur, Maharashtra, over a one-year period, from January to December 2024. The Institutional Ethics Committee approved this research proposal vide letter no. IEC/Pharmac/2025/1607.

Patient enrolment

The study included case records of all inpatients and outpatients with acute febrile illness lasting more than five days who tested positive for chikungunya using the immunoglobulin M (IgM) antibody capture enzyme-linked immunosorbent assay (MAC-ELISA) [4].

Sample collection and processing

A total of 943 patients were tested for chikungunya during the study period, all of whom were recorded and analysed. Serum samples were tested using the IgM Microlisa Assay (an enzyme immunoassay based on the MAC-ELISA technique) according to the manufacturer’s instructions.

Data collection and definitions

Patient data for all samples were retrospectively collected from the hospital management and information system. The study relied on documented follow-up data and did not use patient recall.

Complaints characteristic of chikungunya, such as arthralgia involving small or large joints, skin rash, and vomiting, are recorded as symptoms [4]. Any involvement of a system other than the above symptoms, for example, retro-orbital pain, conjunctivitis, meningitis, respiratory distress, anaemia, and thrombocytopenia, rashes or demonstration of chronicity (persistent arthralgia of more than three months' duration) is recorded as a complication. Any known illness in the patients, like diabetes mellitus, hypertension, hypothyroidism or any immunocompromised condition like sickle cell anaemia, has been recorded under co-morbidities. Any laboratory-confirmed diagnosis of dengue, malaria, scrub typhus or leptospirosis identified during the same period of illness as chikungunya was classified as a co-infection.

Data analysis and statistics

All data were compiled in a Microsoft Excel sheet (Microsoft Corp., Redmond, WA, USA) and analysed using jamovi version 2.7.12 (The jamovi project, Sydney, New South Wales, Australia). Demographic characteristics, symptoms, co-morbidities and co-infections were summarised as numbers and proportions. Categorical variables were compared using Pearson’s chi-square test when the assumptions regarding expected cell frequencies were satisfied (no expected cell count <1 and ≤20% of cells with expected counts <5). When these assumptions were violated, Fisher’s exact test was used, as appropriate. Cramer’s V was reported as the effect-size measure for associations between categorical variables; a p-value <0.05 was considered significant.

## Results

In this study, a total of 943 non-repetitive samples were tested for chikungunya IgM antibodies, of which 117 (12.4%) samples were positive. The total positive cohort consisted predominantly of adults aged 19-49 years (n = 72, 61.5%), followed by individuals over 50 years (n = 27, 23.1%), and those under 18 years of age (n = 18, 15.4%). The sex ratio of the study population was 1.1:1. A majority of patients resided in urban areas (n = 71, 60.7%). The demographic data are tabulated in Table 1.

**Table 1:** Demographic characteristics of chikungunya-positive patients (N = 117)

Characteristics		Frequency (%)
Age (in years)	<18	18 (15.4)
	19-49	72 (61.5)
	>50	27 (23.1)
Sex	Male	60 (51.3)
	Female	57 (48.7)
Area	Urban	71 (60.7)
	Rural	46 (39.3)

All lab-confirmed seropositive patients (n = 117) presented with fever lasting more than five days. Large joint involvement includes arthralgia of proximal joints such as the shoulders, elbows, spinal joints, and knees and/or hips. All joints distal to the above-mentioned were considered under small joint involvement (e.g. wrist, joints of fingers, ankle, joints of feet). Arthralgia was the predominant clinical symptom, with large-joint involvement reported in 77 (65.8%) patients and small-joint involvement in 59 (50.4%) patients. The classical triad of fever, rash, and arthralgia was observed in 24 (20.5%) cases. Among the comorbid conditions, diabetes mellitus was the most prevalent (n = 10, 8.5%), followed by hypertension (n = 8, 6.8%). Co-infections were uncommon, with dengue being the most frequently identified (n = 4, 3.4%).

Fewer than half of the patients (n = 51, 43.6%) developed complications. Among all age groups, the working population aged 19-49 years had slightly lower rates of complications than children and the elderly. There was a slightly higher percentage of complications in females (49.1%) than in males (38.3%), and in patients from rural settings (50.0%) than in those from urban settings (39.4%).

Among all complications, persistent arthralgia beyond three months was the most frequently reported (n = 16, 31.4%). Another complication was joint swelling (n = 8, 15.7%). Neurological features were rare: meningitis occurred in three patients (5.9%), and peripheral nerve involvement in one (1.9%). Only one death was recorded in our cohort. The different types of complications recorded are depicted in Figure 1.

**Figure 1:**
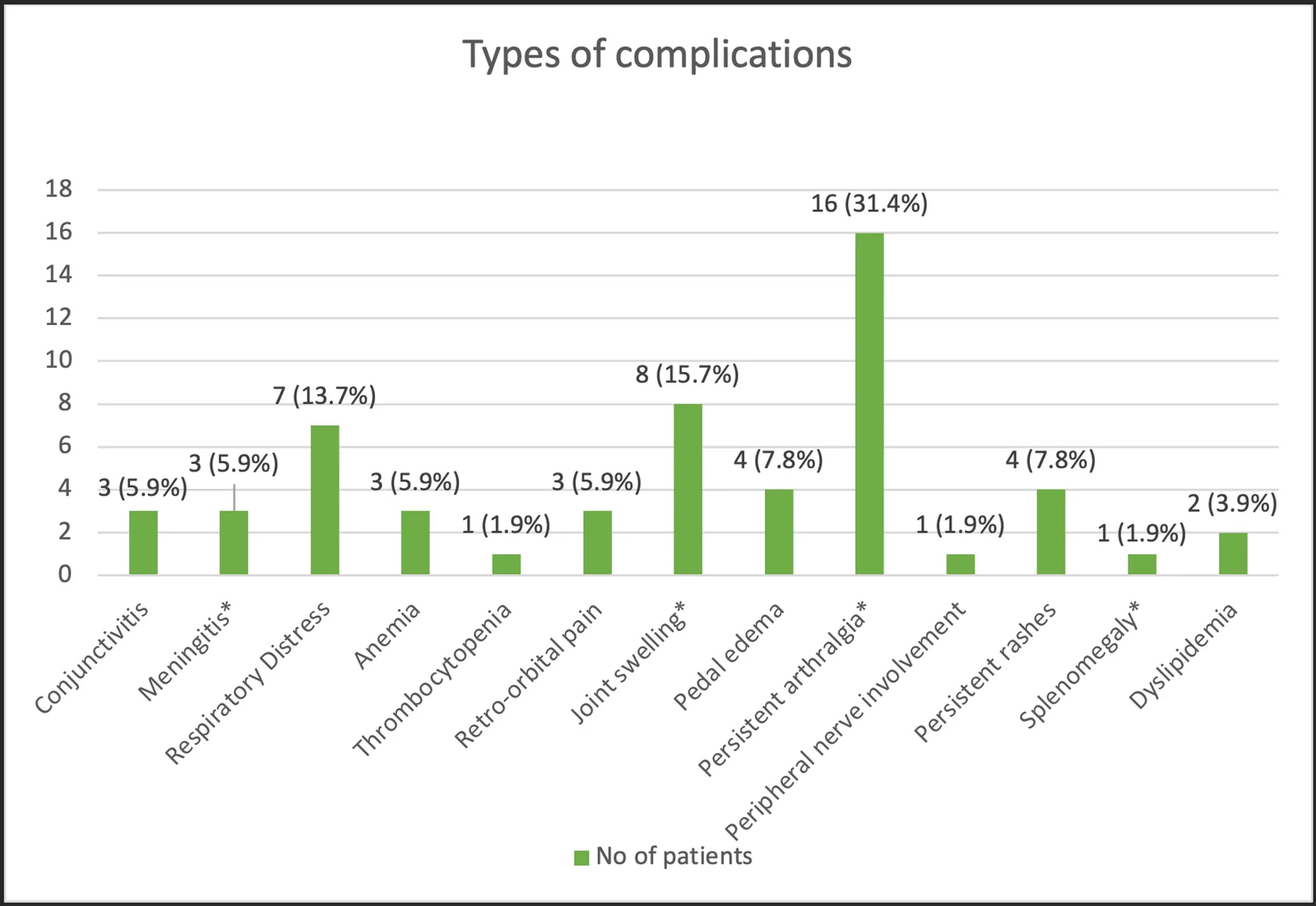
Different types of complications recorded (n = 51) * Patients with multiple complications were included in more than one category.

The comparison of demographics, symptoms, comorbidities, and co-infections with the development of complications has been calculated and depicted in Table 2.

**Table 2:** Comparison of demographic characteristics, symptoms, comorbidities and co-infections with development of complications (N = 117) ^ Only female patients were included in the comparison of antenatal care (ANC) status and complications. ^* ^Fisher's exact test was used instead of the chi-square test because the assumptions for the chi-square test were violated. ^#^ Patients with both large- and small-joint involvement were included in both categories.

Characteristics		Total	Complications present	Chi-square statistics			
		(N = 117), n	(n = 51), n (%)	χ^2^	p-value	df	Cramér's V
Age (in years)	<18	18	9 (50.0)	0.850	0.654	2	0.085
	19-49	72	29 (40.3)				
	>50	27	13 (48.1)				
Sex	Male	60	23 (38.3)	1.384	0.239	1	0.109
	Female	57	28 (49.1)				
Area	Urban	71	28 (39.4)	1.267	0.260	1	0.104
	Rural	46	23 (50.0)				
Symptoms							
Large joint pain^#^	Yes	77	33 (42.9)	0.049	0.825	1	0.021
	No	40	18 (45.0)				
Small joints^#^	Yes	59	25 (42.4)	0.072	0.789	1	0.025
	No	58	26 (44.8)				
Vomiting	Yes	13	5 (38.5)	-	0.773*	-	-
	No	104	46 (44.2)				
Rash	Yes	31	14 (45.2)	0.042	0.837	1	0.019
	No	86	37 (43.0)				
Comorbidities							
Diabetes mellitus	Yes	10	5 (50.0)	-	0.745*	-	-
	No	107	46 (43.0)				
Hypertension	Yes	8	5 (62.5)	-	0.293*	-	-
	No	109	46 (42.2)				
Hypothyroidism	Yes	4	3 (75.0)	-	0.316*	-	-
	No	113	48 (42.5)				
Asthma	Yes	3	3 (100.0)	-	0.080*	-	-
	No	114	48 (42.1)				
Tuberculosis	Yes	1	1 (100.0)	-	0.436*	-	-
	No	116	50 (43.1)				
Sickle cell anaemia	Yes	3	1 (33.3)	-	1.000*	-	-
	No	114	50 (43.9)				
Transplant donor	Yes	1	1 (100.0)	-	0.436*	-	-
	No	116	50 (43.1)				
ANC^ (n = 57)	Yes	3	2 (66.7)	-	0.611*	-	-
	No	54	26 (48.2)				
Co-infections							
Dengue	Yes	4	3 (75.0)	-	0.316*	-	-
	No	113	48 (42.5)				
Malaria	Yes	1	0 (0.0)	-	1.000*	-	-
	No	116	51 (44.0)				

## Discussion

CHIKV has been circulating in India since the early 1950s, and the first outbreak was recorded in 1963 [7]. Our study reported 12.4% confirmed cases in 2024. The majority of the chikungunya-positive cases were in the working-age group (61.5%), a finding similarly reported in studies by Joshi et al. [8], Ozair et al. [9] and Ray et al. [10]. The ratio of male to female chikungunya-positive cases was nearly equal, as it is known to be transmitted to males and females equally [11]. Reshma et al. [12], Ozair et al. [9], and Sagar et al. [13] have reported similar findings from Indian data, and Imad et al. [14] and Anwar et al. [15] from data of other South Asian countries. In tropical and temperate countries, CHIKV is known to be transmitted and maintained in an urban cycle [11]. Similarly, in our study, the most positive cases were from urban settings compared to rural settings.

Joint pain is the defining clinical feature of CHIKV infection and is commonly used to distinguish it from other arboviral infections in endemic settings like India [5]. This was also evident in our study, as arthralgia was the most common symptom. Chikungunya is known to affect the smaller joints more frequently than the larger proximal joints, such as the knee [4]. The literature review [16-19] has shown that the most commonly affected joints in chikungunya are the finger, wrist, and ankle joints. However, in our study, involvement of the large joints (65.8%) was more common than that of the smaller joints (50.4%). Cutaneous manifestations of the disease often begin with flushing over the face and trunk, followed by the appearance of macular and papular lesions on the trunk and limbs [5]. Even though it is part of the classical triad, the frequency of rash varies widely in the literature. Reshma et al. [12] reported a low occurrence of 25.7%, while Liu et al. [20] reported 48.2%, and Chang et al. [16] reported rash in as many as 85% of chikungunya patients. In our study, rash was seen in only 26.5% of patients. The classical triad of fever, arthralgia and rash seen in chikungunya was reported in 20.5% of cases. According to the literature [20,21], 31.6-48.21% of patients present with the classical triad, a proportion higher than in our study. Vomiting was seen in very few cases (11.1%). This is much lower than in other studies such as those by Ray et al. [10], Sagar et al. [13], and Reshma et al. [12], which were conducted in 2009, 2016, and 2019, respectively.

The majority of patients (56.4%) did not develop complications and recovered completely. A total of 43.6% of patients developed complications. The most common complication documented in our study is persistent arthralgia (31.4%), i.e., arthralgia that continues after the acute stage of the illness has resolved, often for more than three months [4]. This rate is similar to the study by Kularatne et al. [22], where they reported 45% chronic arthritic disability in their cohort. While most symptoms resolve within approximately one week, joint pain may last for several weeks to months. This prolonged joint pain associated with CHIKV is believed to result from the continued presence of viral antigens in the joints, leading to a sustained inflammatory response [5]. The second most common complication was joint swelling (15.7%). It is similar to the data reported by Ray et al. [10]; however, Amin et al. [21] reported a higher rate of joint swelling (49.3%).

Incidentally, the only death reported in our study was of a patient succumbing to respiratory distress, although the root cause of death cannot be attributed to chikungunya. Occasional cases were reported, including conjunctivitis (5.9%), anaemia (5.9%), thrombocytopenia (1.9%), persistent hyperpigmented rashes (7.8%), splenomegaly (1.9%), dyslipidaemia (3.9%), and retro-orbital pain (5.9%). Neurological manifestations, albeit rare [4], have been increasingly reported.

The patient demographics, presence and absence of symptoms, comorbidities and co-infections have been compared with the development of complications using the chi-square test and Fisher's exact test when more than 20% of the cells had an expected count of <5. There is a slight female preponderance among those who develop complications (p = 0.239). The presence of all other symptoms and comorbidities is associated with a slightly higher rate of complication development, although none are significantly higher. The presence of asthma, co-infection with tuberculosis and being a transplant donor showed a higher proportion of complications; however, on applying Fisher’s exact test, the association was not statistically significant.

The study has limitations such as asymptomatic or mild cases without fever not being included, so the study population may not be representative of all chikungunya infections; as it is a single-centre study, selection bias might be present; molecular testing has not been done. Therefore, there is a possibility of false-positive tests due to cross-reactivity; another limitation is the absence of a comparator group; and the study was not powered for predictor analysis, and the limited number of outcome events precluded reliable multivariable adjustment. Therefore, the reported associations are unadjusted and should be interpreted accordingly.

## Conclusions

The seropositivity study of the CHIKV upsurge has provided valuable insights into the symptomatology and complications associated with chikungunya infection. Demographically, chikungunya affects all age groups. Arthralgia is the most commonly recorded symptom. The study observed a lower frequency of rash and vomiting in the cohort. Chronic arthralgia for more than three months is the most common complication seen. Although studies have described greater involvement of small joints than large joints, in our case, we have reported a higher number of large-joint involvements in chikungunya. Following the acute phase, patients should be followed up for persistent chronic arthralgia and joint swelling.
